# Live imaging of endogenous protein dynamics in zebrafish using chromobodies

**DOI:** 10.1242/dev.118943

**Published:** 2015-05-15

**Authors:** Paolo Panza, Julia Maier, Christian Schmees, Ulrich Rothbauer, Christian Söllner

**Affiliations:** 1 Max-Planck-Institut für Entwicklungsbiologie, Abteilung Genetik, Spemannstraße 35, Tübingen 72076, Germany; 2 Naturwissenschaftliches und Medizinisches Institut der Universität Tübingen, Markwiesenstraße 55, Reutlingen 72770, Germany; 3 Pharmazeutische Biotechnologie, Eberhard Karls Universität Tübingen, Auf der Morgenstelle 8, Tübingen 72076, Germany

**Keywords:** Actin, PCNA, Live imaging, Nanobody, Protein dynamics

## Abstract

Chromobodies are intracellular nanoprobes that combine the specificity of antibodies with the convenience of live fluorescence imaging in a flexible, DNA-encoded reagent. Here, we present the first application of this technique to an intact living vertebrate organism. We generated zebrafish lines expressing chromobodies that trace the major cytoskeletal component actin and the cell cycle marker PCNA with spatial and temporal specificity. Using these chromobodies, we captured full localization dynamics of the endogenous antigens in different cell types and at different stages of development. For the first time, the chromobody technology enables live imaging of endogenous subcellular structures in an animal, with the remarkable advantage of avoiding target protein overexpression or tagging. In combination with improved chromobody selection systems, we anticipate a rapid adaptation of this technique to new intracellular antigens and model organisms, allowing the faithful description of cellular and molecular processes in their dynamic state.

## Abstract (precis)

**SUMMARY:** Chromobodies - small, intracellular fluorescent antibodies - are used to trace endogenous antigens, without the need for direct protein tagging, in zebrafish embryos.

## INTRODUCTION

Nanobodies are single-domain IgG fragments derived from heavy-chain antibodies found in the serum of *Camelidae* (Hamers-Casterman et al., 1993). Being the smallest antigen-recognizing protein elements known to date, they offer several advantages as research tools (reviewed by Muyldermans, 2013): nanobodies can easily be cloned from naïve or immunized animals and screened for antigen binding by phage display or targeted immunoaffinity (Arbabi Ghahroudi et al., 1997; Fridy et al., 2014). They vary in their target-binding affinities, allowing the isolation of strong and weak binders for different applications. Their small size (∼12-15 kDa) allows antigen recognition even in cases where accessibility is limited for conventional antibodies, which are about ten times larger than nanobodies (Muyldermans, 2013). Nanobodies preferably bind three-dimensional epitopes presented by intact antigens organized in multiprotein complexes. Chimeric constructs comprising the binding moiety of nanobodies fused to fluorescent proteins (referred to as chromobodies) have been successfully employed to visualize endogenous intracellular antigens in mammalian cells (Rothbauer et al., 2006; Kaiser et al., 2014). Stable expression of these intracellular antibodies has been shown to be well tolerated by various cell types, reliably tracing targeted antigens in diverse cellular compartments (Burgess et al., 2012; Helma et al., 2012; Rothbauer et al., 2006; Zolghadr et al., 2012). These observations introduce the possibility of exploiting the antigen-tracing capabilities of chromobodies in intact organisms, while circumventing direct tagging or overexpression of the target protein, which are known causes of localization artefacts (Huh et al., 2003; Swulius and Jensen, 2012). However, until now, chromobodies have not been employed to describe cellular and developmental processes in living animals.

Here, we present the first application of the chromobody technology in a vertebrate model organism. To achieve this, we took advantage of the zebrafish, in which cellular and molecular processes can be followed by live imaging over the early stages of development.

In order to analyse the potential of chromobodies to trace endogenous proteins in zebrafish, we focused on actin and on the proliferating cell nuclear antigen (PCNA). Both proteins display characteristic dynamic localization patterns in different subcellular compartments. Their essential roles in fundamental cellular functions, like motility and proliferation, make these antigens attractive candidates for establishing the chromobody technology *in vivo* and assessing its impact on protein function. By generating transgenic lines expressing recently described actin- and PCNA-binding chromobodies (Burgess et al., 2012; Rocchetti et al., 2014; Akopyan et al., 2014; Kaiser et al., 2014), we successfully followed the subcellular localization of these proteins in complex developing tissues over time.

## RESULTS AND DISCUSSION

**Fig. 1.**
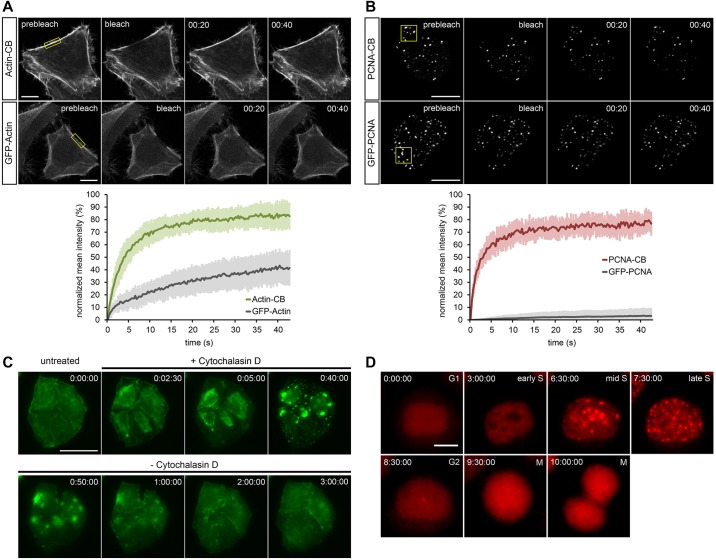
**Localization dynamics of actin-CB and PCNA-CB in HeLa cells.** (A) FRAP of actin-CB (upper row) or GFP-actin (lower row) transiently expressed in HeLa cells. Photobleaching of a small region (yellow box) shows a significantly faster recovery (t_1/2_: 3.83 s) of actin-CB compared with GFP-actin, indicating transient antigen binding. The plot shows average values of total fluorescence at consecutive time points, *n*=10. Error bars indicate s.d. (B) Photobleaching of replication foci during S phase (yellow box) shows fast recovery of the PCNA-CB signal (t_1/2_: 1.81 s), whereas almost no recovery of GFP-PCNA can be detected. The plot shows average values of total fluorescence at consecutive time points, *n*=10. Error bars indicate s.d. (C) HeLa cells stably expressing actin-CB imaged upon treatment with 2 μM cytochalasin D (an actin polymerization inhibitor) for 40 min and during subsequent recovery (180 min). Time-lapse imaging reveals drug-induced actin reorganization. (D) Time-lapse analysis of a HeLa cell stably expressing PCNA-CB. During G1, the chromobody signal is evenly distributed throughout the nucleus. Over time, granular foci redistribute at sites of DNA replication, indicating the progression of S phase (3-7.5 h), until foci disappear in G2 (8.5 h) and the cell divides (10 h). Time-stamps: min:s (A,B), h:min:s (C,D). Scale bars: 10 μm in A,B,D; 50 μm in C.

Cell lines that stably express chromobodies against the major component of the cellular cytoskeleton, F-actin, and against human PCNA have recently become available (Burgess et al., 2012; Rocchetti et al., 2014; Akopyan et al., 2014; Kaiser et al., 2014). In our first approach, we used HeLa cells stably expressing either chromobody to visualize the localization dynamics of the corresponding antigens in real time. To analyse their intracellular binding properties, we performed FRAP (fluorescence recovery after photobleaching) experiments. Both chromobodies show significantly faster recovery after photobleaching compared with their fluorescently labelled antigens (GFP-actin and GFP-PCNA) in cells (Fig. 1A,B). These data are indicative of a large mobile chromobody fraction composed of highly diffusible molecules. Furthermore, immediately after bleaching, we observed the relocalization of chromobodies to cellular structures that were marked in the prebleaching condition. These results suggest a transient but specific antigen-binding mode, which is characterized by a high on-rate combined with a high off-rate, for both chromobodies in living cells. We therefore hypothesized that this reversibility in binding can minimize any interference these chromobodies might exert on target protein function. In agreement with our findings, we could successfully visualize detailed cytoskeletal remodelling after incubation with F-actin-modulating compounds (Fig. 1C). Similarly, PCNA chromobodies recapitulate the dynamics of endogenous PCNA throughout the cell cycle (Fig. 1D). This is in accordance with previous findings, showing that the expression of chromobodies in eukaryotic cells does not interfere with cell cycle progression (Burgess et al., 2012) or formation of actin filaments (Plessner et al., 2015; Rocchetti et al., 2014). Based on these results, we asked whether the chromobody technology is applicable to living organisms such as zebrafish.

**Fig. 2.**
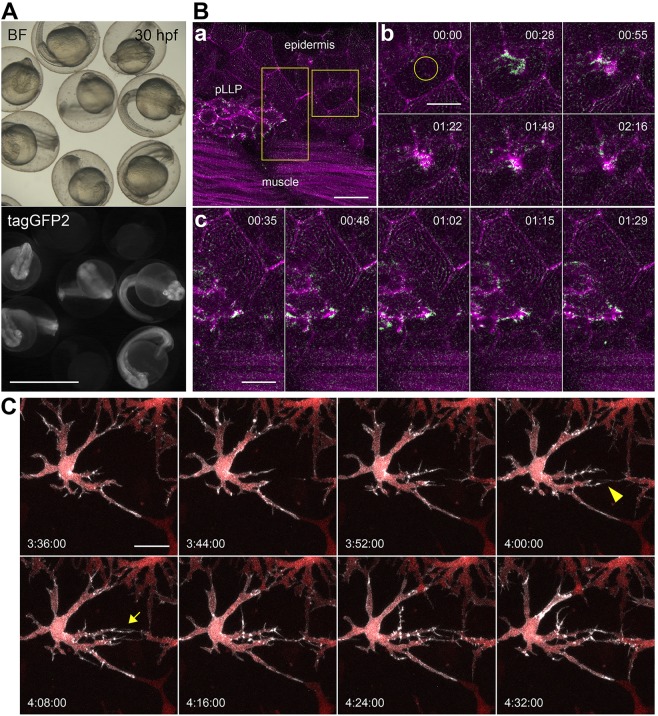
**Actin-CB-expressing transgenic zebrafish embryos reveal fast actin dynamics in multiple cell types.** (A) 30-hpf embryos from an outcross of *hsp70l:Actin-CB* founder fish. The embryos were heat-shocked at 24 hpf. Chromobody-expressing individuals show widespread fluorescence and no morphological abnormalities when compared with non-transgenic siblings. BF, brightfield. (B) Actin-CB can trace fast protein dynamics in living zebrafish. (a) Overview of a 36 hpf embryo, in which actin-CB expression was induced by heat-shock at 24 hpf. pLLP cells are visible, together with epidermal cells and muscle fibres. (b) Reorganization of actin after laser-induced wounding (yellow circle). (c) Detailed actin activity at the leading edge of front cells during pLLP migration. Magenta indicates signal from time frame *t*+1. Green indicates signal obtained from the subtraction of frame *t* from frame *t*+1. Green signal highlights the appearance of actin-CB signal from frame to frame (scanning interval is 7 s). (C) Filopodial activity during establishment of intercellular contacts between neighbouring xanthophores. Detail of a *csf1ra:gal4* embryo expressing *UAS:NTR-mcherry* (red) and *UAS:Actin-CB* (white). Filopodia extruded from xanthophore branches are directional to the prospective contact site with neighbouring cells (arrowhead and arrow). Time-stamps: min:s (B), h:min:s (C). Scale bars: 1 mm in A; 10 μm in Bb,c; 20 μm in Ba,C.

With the aim of having temporal control over chromobody expression at any developmental stage, we generated zebrafish transgenic lines expressing the actin chromobody as a tagGFP2 fusion protein (actin-CB) and the PCNA chromobody as a tagRFP fusion protein (PCNA-CB) under the control of the well-characterized *hsp70l* promoter (Halloran et al., 2000). By incubating transgenic embryos carrying either *hsp70l:Actin-CB* or *hsp70l:PCNA-CB* at 39°C for 1 h, we successfully induced chromobody expression at 24 hpf (Fig. 2A; supplementary material Fig. S1) and at 48 hpf (data not shown) with similar effects. Fluorescent signal was widespread in the embryo and marked different cell types, distributed throughout the body (Fig. 2A,Ba). Notably, embryos tolerate the strong and ubiquitous expression of both chromobodies after heat-shock induction and can be raised to adulthood, suggesting that these molecules do not significantly interfere with normal animal development (Fig. 2A; supplementary material Fig. S1).

In order to validate the specificity of our chromobody-based zebrafish lines, we examined the degree of colocalization between actin-CB and F-actin, as detected by phalloidin staining. In addition, we counterstained embryos expressing PCNA-CB with anti-PCNA antibodies. In both instances we detected comparable fluorescence patterns between the chromobodies and their respective antigens (supplementary material Figs S2 and S3), indicating that the chromobodies, which were selected against human actin and PCNA, recognize the orthologous zebrafish proteins and recapitulate their endogenous cellular distribution. Constitutive expression of chromobodies may, however, result in a fluorescent background derived from diffusively distributed, unbound molecules. This can be seen as a weak cytoplasmic haze in chromobody-expressing HeLa cells, as well as occasionally in transgenic embryos. Next, by injecting a plasmid expressing human PCNA-GFP (Leung et al., 2011) in *hsp70l:PCNA-CB* embryos, we observed that exogenous, fluorescently labelled human PCNA and PCNA-CB assemble in the same nuclear structures during S phase and dynamically colocalize (supplementary material Fig. S4 and Movie 4).

To test whether chromobodies reproduce the localization dynamics of actin *in vivo*, we induced actin-CB expression in 24-hpf embryos and analysed different cell types and processes after 4-6 h. We detected intense fluorescence in embryonic muscle fibres and in epidermal cells, including their characteristic actinic apical ridges (Fig. 2Ba). After inducing a wound in the epidermis, we observed rapid relocalization of the cellular actin to the damage site, followed by slow reabsorption of the actin accumulation (Fig. 2Bb).

The posterior lateral line primordium (pLLP) is a cranial ganglionic structure that migrates from 22 hpf along the horizontal myoseptum towards the tip of the tail (Kimmel et al., 1995). Along its way, the primordium deposits trailing mechanosensory organs: the neuromasts. By monitoring the chromobody signal, we were able to trace fast actin dynamics at the leading edge of cells in the migrating primordium (Fig. 2Bc; supplementary material Movies 1,5). These data show that actin-directed chromobodies do not interfere with the overall directionality of the primordium, although its migration crucially depends on actin-based filopodial activity at the margin (Xu et al., 2014).

To take fully advantage of the combinatorial expression opportunities in zebrafish, we next integrated chromobody expression with the Gal4/UAS system. Because in our transgenic lines chromobody transcription is controlled by upstream activating sequences (UAS), they can be readily combined with the large number of Gal4 drivers established in zebrafish (Bussmann and Schulte-Merker, 2011; Kawakami et al., 2010; Levesque et al., 2013).

*UAS:Actin-CB* founder fish were tested by using a *csf1ra:gal4* driver line (Gray et al., 2011). These fish express Gal4 in macrophages and xanthophores, a pigmented cell type. Macrophages are fast migratory cells that continuously patrol the body in search of inflammatory cues and physical damage. By contrast, xanthophores are large, flat, relatively static cells that display dynamic membrane protrusions, and hence are suitable for analysis of actin dynamics. In both cell types, actin-CB signal is localized primarily at sites of the plasma membrane. Time-lapse analysis of xanthophores revealed filopodial activity preceding the formation of intercellular contacts mediated by these cells. Importantly, before neighbouring cell protrusions were in contact with each other, filopodia projected directionally towards the future contact site (arrowhead and arrow in Fig. 2C; supplementary material Movie 2). This behaviour suggests that xanthophores can sense neighbouring contact partners, probably through extracellular cues. In conclusion, by following the chromobody signal we were able to visualize fast actin dynamics, as well as to describe the rearrangement of actinic cytoskeletal elements over longer developmental periods.

**Fig. 3.**
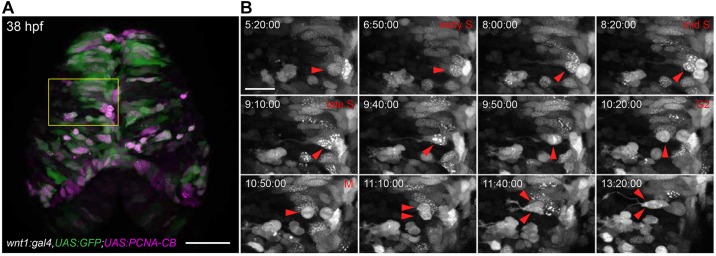
**Cell cycle analysis of transgenic zebrafish embryos expressing PCNA-CB.** (A) Overview of the dorsal midbrain of a *wnt1:gal4,UAS:GFP* (green); *UAS:PCNA-CB* (magenta) double transgenic embryo at 38 hpf. (B) Unaltered cell cycle progression in PCNA-CB-expressing, *wnt1*-positive neural progenitors. PCNA-CB signal transitions from a speckled configuration (S phase) to an evenly distributed one (G2), which precedes mitosis (M). Arrowheads indicate a cell progressing through its last cycle before terminally differentiating into two daughter neurons. Time-stamps: h:min:s. Scale bars: 50 μm in A; 20 μm in B.

To reveal the subcellular localization of PCNA in actively dividing cells, we crossed *UAS:PCNA-CB* founder fish to a *wnt1* driver line (Volkmann et al., 2010). In these transgenic embryos, the dorsal midbrain is sparsely but strongly labelled during the first few days of development (Fig. 3A). The high level of transactivation induced by the *wnt1* promoter fragment was instrumental in testing for possible cellular toxicity caused by the expression of chromobodies. We did not detect obvious signs of cellular stress or macroscopic cell death in these embryos. We reasoned that if PCNA-directed chromobodies can interfere with constitutive PCNA-dependent DNA replication, delayed neurogenesis and consequent morphological defects would be observed. By contrast, embryos that express PCNA-CB from 16 hpf were healthy and could be raised to adulthood. Live imaging conducted from 30 hpf to ∼42 hpf revealed that neighbouring cells were undergoing asynchronous cell cycles, as they showed distinct PCNA-CB localization patterns. We focused further analysis on cells displaying finely speckled chromobody signal in their nuclei, a mark of S phase. Over time, foci gradually decreased in number and increased in size, indicating the progression from early to late S phase, during which heterochromatin is replicated (Burgess et al., 2012). Immediately afterwards, a dramatic localization shift from speckles to uniform nuclear labelling was recorded shortly before the cells underwent mitosis (arrowheads in Fig. 3B; supplementary material Movies 3,6). These observations specifically mimic the reported cell cycle-dependent localization pattern of PCNA in mammalian cells (Leonhardt et al., 2000; Essers et al., 2005). After cell division, the chromobody relocalizes to the cytoplasm, from where PCNA is actively transported back into the nucleus during G1.

Thus, our data demonstrate that PCNA chromobodies faithfully monitor the cell cycle progression through S phase (nuclear densities) to G2 (uniform distribution in the nucleus) and M phase with high fidelity in neural progenitors. Compared with other cell cycle indicators, such as the FUCCI system (Sugiyama et al., 2009; Sakaue-Sawano et al., 2008), PCNA-CB directly reports cell cycle-dependent shifts in protein localization. Our simpler method achieves greater resolution during S phase and can differentiate between S and G2 phases without the need to quantify fluorescence levels.

In the past two decades, zebrafish has emerged as an ideal model with which to follow cellular and molecular processes over the first days of development, especially by means of live cell imaging. Given the limited number of targeted GFP knock-in lines available, however, the study of protein dynamics has traditionally relied on the expression of fluorescent fusion proteins from additional gene copies. With the introduction of TALEN- and CRISPR/Cas9-based genome editing methods (Cermak et al., 2011; Jinek et al., 2012), targeted knock-in of fluorescent tags has now been demonstrated (e.g. Dickinson et al., 2013). The application of this technology to protein tagging, however, is still emerging. Despite the ease of modern gene editing tools, analyses of overexpressed and/or tagged proteins frequently fail to recapitulate the endogenous protein characteristics, such as expression level, turnover, functional epitope availability and, as a consequence, their localization and dynamics (Huh et al., 2003; Swulius and Jensen, 2012; Khmelinskii et al., 2012).

An additional obstacle to protein localization description in zebrafish is the very limited availability of antibody reagents. Antibodies that are already developed for orthologous proteins, especially from mouse or human, rarely crossreact in zebrafish. Moreover, concern has been expressed regarding the impact of fixation/permeabilization procedures on antigen localization, such that comparison with live imaging data has been recommended (Schnell et al., 2012).

Here, we present the first application of the chromobody technology to a vertebrate organism. By creating zebrafish transgenic lines expressing intracellular actin- and PCNA-binding chromobodies, we can now follow the subcellular localization of these endogenous proteins in complex developing tissues. Notably, the development of zebrafish embryos is unaffected even in the presence of highly expressed chromobodies throughout different embryonic stages. Our approach does not rely on target protein overexpression or tagging, and it is therefore less likely to interfere with protein function.

Taken together, our data show that chromobody-based antigen tracing in zebrafish can be expanded to different target proteins and might be extended to other organisms. We introduce our transgenic lines as a valuable resource for molecular phenotyping or disease modelling, e.g. monitoring proliferation in cancer cells and high-content drug screening.

Together with improved nanobody generation and selection techniques (Fridy et al., 2014) that have emerged recently, our work sets the stage for future large-scale adoption and optimization of chromobody-based genetically encoded reagents for zebrafish, among other model organisms.

## MATERIALS AND METHODS

### Expression plasmids

Actin-CB-TagGFP2, PCNA-CB-TagGFP2 and PCNA-CB-TagRFP plasmids were kindly provided by ChromoTek. GFP-β-actin (Clontech) and GFP-PCNA (Leonhardt et al., 2000) were also used.

### Mammalian cell culture and transfection

HeLa cells stably expressing PCNA-CB-TagRFP or actin-CB-TagGFP2 were cultivated according to standard protocols, using DMEM containing high glucose, pyruvate, 10% foetal calf serum, L-glutamine and antibiotics. Cells were trypsinized for passaging and cultivated at 37°C in a humidified chamber with a 5% CO_2_ atmosphere. Plasmid transfections using Lipofectamine 2000 (Life Technologies) were carried out according to the manufacturer's instructions.

### Fluorescence recovery after photobleaching (FRAP)

HeLa cells were seeded in a µ-slide 8-well chamber (Ibidi) and transiently transfected with GFP-actin, actin-CB-TagGFP2, GFP-PCNA or PCNA-CB-TagGFP2. FRAP recordings were performed with a Zeiss LSM 510 laser scanning confocal microscope using a 488 nm argon laser. This was set to 50% output and 100% transmission to photobleach a region of interest for 1.7 s. Confocal series were acquired using 1% laser transmission with the pinhole opened to 1.5 Airy units. Five prebleach and 145 postbleach images were recorded at maximum speed (294 ms intervals). Normalized mean fluorescence intensities were corrected for background and for total loss of fluorescence over time. Fluorescence recovery curves were fitted with Origin 7.5 (OriginLab) using an exponential function, given by *I*(*t*)=*A*(1−*e*^−*kt*^), where *I*(*t*) is the signal intensity dependent on time, *A* is the end value of intensity, *k* is the time constant. Half-times of recovery were determined by *t*_1/2_=*ln* 0.5/−*k*.

### Time-lapse analysis

Stable HeLa cell lines (Actin-CB-tagGFP2, PCNA-CB-tagRFP) were seeded in µClear 96-well plates (Greiner) and recorded with an Image Xpress micro XL system (Molecular Devices). Actin-CB-tagGFP2 cells were treated with 2 µM cytochalasin D (Sigma-Aldrich) for 40 min and imaged at 2.5 min intervals, followed by a change to Cytochalasin D-free medium and additional imaging for 140 min (with 10 min intervals). Live imaging of PCNA-CB-tagRFP cells was carried out for 24 h at 1 h intervals.

### Zebrafish lines and animal maintenance

All zebrafish experiments were performed in accordance with the guidelines of the Max Planck Society and approved by the Regierungspräsidium Tübingen (Aktenzeichen: 35/9185.46). The following strains were used and reared as described previously (Nüsslein-Volhard and Dahm, 2002): wild-type Tübingen, *Tg(hsp70l:Actin-CB,cmlc2:GFP)*, *Tg(hsp70l:PCNA-CB,cmlc2:GFP)*, *Tg(UAS:Actin-CB,crybb:eCFP)*, *Tg(UAS:PCNA-CB,crybb:eCFP)*, *TgBAC(csf1ra:Gal4-VP16)^i186^* and *Tg(UAS-E1b:NTR-mCherry)^i149^* (Gray et al., 2011), and *Tg(-6.6wnt1:Gal4-VP16-6.7wnt1,14xUAS-E1b:EGFP)^tg2229^* (Volkmann et al., 2010).

### Generation of zebrafish transgenic lines

Actin-CB and PCNA-CB were cloned into pDONR221 (Invitrogen) to generate Gateway-compatible Middle Entry (pME) constructs. These were recombined with Tol2kit plasmids (Kwan et al., 2007) p5E-*hsp70*, p3E-polyA and pDEST-Tol2CG2 to generate *HS:Actin-CB* and *HS:PCNA-CB* constructs. Actin-CB and PCNA-CB entry clones were recombined with p5E-UAS, p3E-polyA and pDEST-Tol2-*crybb:eCFP* (a gift from Darren Gilmour, EMBL, Heidelberg, Germany) to generate *UAS:Actin-CB* and *UAS:PCNA-CB* constructs. Gateway recombinations were performed according to the Tol2kit instructions (http://tol2kit.genetics.utah.edu/). The obtained constructs were co-injected at 10 ng/μl with 30 ng/μl Tol2 transposase mRNA. Nucleic acid solution (1-2 nl) was injected into one-cell stage embryos. Embryos were sorted for the expression of the transgenesis marker and raised to adulthood. Founders were identified during outcrosses and their transgenic progenies were raised independently.

### Antibody and phalloidin staining

*hsp70l:Actin-CB* and *hsp70l:PCNA-CB* embryos were heat-shocked at 24 hpf and fixed at 36 hpf. *hsp70l:Actin-CB* embryos were incubated in 1:500 rhodamine phalloidin (Molecular Probes R415) for 30 min, followed by several washes in PBST (PBS+0.1% Triton X-100). *hsp70l:PCNA-CB* embryos were used in conventional immunostaining. In order to detect endogenous PCNA, rabbit polyclonal anti-PCNA (GeneTex, GTX124496) and Alexa 488 goat anti-rabbit IgG (Molecular Probes, A-11008) were used at 1:200 and 1:400 dilutions, respectively.

### PCNA-GFP/PCNA-CB analysis

A pCS2+ based human PCNA-GFP plasmid (1-2 nl of a 10 ng/μl solution) (a gift from Caren Norden, MPI-CBG, Dresden, Germany) was injected into one-cell stage *hsp70l:PCNA-CB* embryos. All embryos were heat-shocked at 24 hpf. Animals sorted for GFP and tagRFP expression were used in live imaging experiments beginning at 36 hpf.

### Live imaging

Transgenic embryos at different stages were manually dechorionated and mounted on glass-bottom petri dishes (35 mm, MatTek) using 0.8% low-melting-point agarose. During imaging sessions, they were submerged in E2 medium supplemented with 0.003% PTU to inhibit melanogenesis (Nüsslein-Volhard and Dahm, 2002). A Zeiss LSM 780 NLO confocal microscope was used to acquire scans. For wound induction, an 800 nm wavelength MaiTai laser was used at 100% intensity with a 0.41 μs pixel dwell time. Data processing and analysis were carried out using Fiji (Schindelin et al., 2012) and Zeiss ZEN 2010 software.

## Supplementary Material

Supplementary Material
