# Supplementary material for: Live imaging of endogenous protein dynamics in zebrafish using chromobodies
Source: Development. 2015 May 15;142(10):1879–84. doi: 10.1242/dev.118943 (PMC4440926; doi:10.1242/dev.118943)
Supplement: Supplementary Material [file supp_142_10_1879__index.html]

Supplementary Material 

# Live imaging of endogenous protein dynamics in zebrafish using chromobodies

## DEV118943 Supplementary Material

- Supplementary Material
