# Supplementary material for: Live imaging of endogenous protein dynamics in zebrafish using chromobodies
Source: Development. 2015 May 15;142(10):1879–84. doi: 10.1242/dev.118943 (PMC4440926; doi:10.1242/dev.118943)
Supplement: Supplementary Material [file supp_142.10.1879_DEV118943supp.pdf]

Panza et al. - Live imaging of endogenous protein dynamics in zebrafish using chromobodies

Supplementary Information

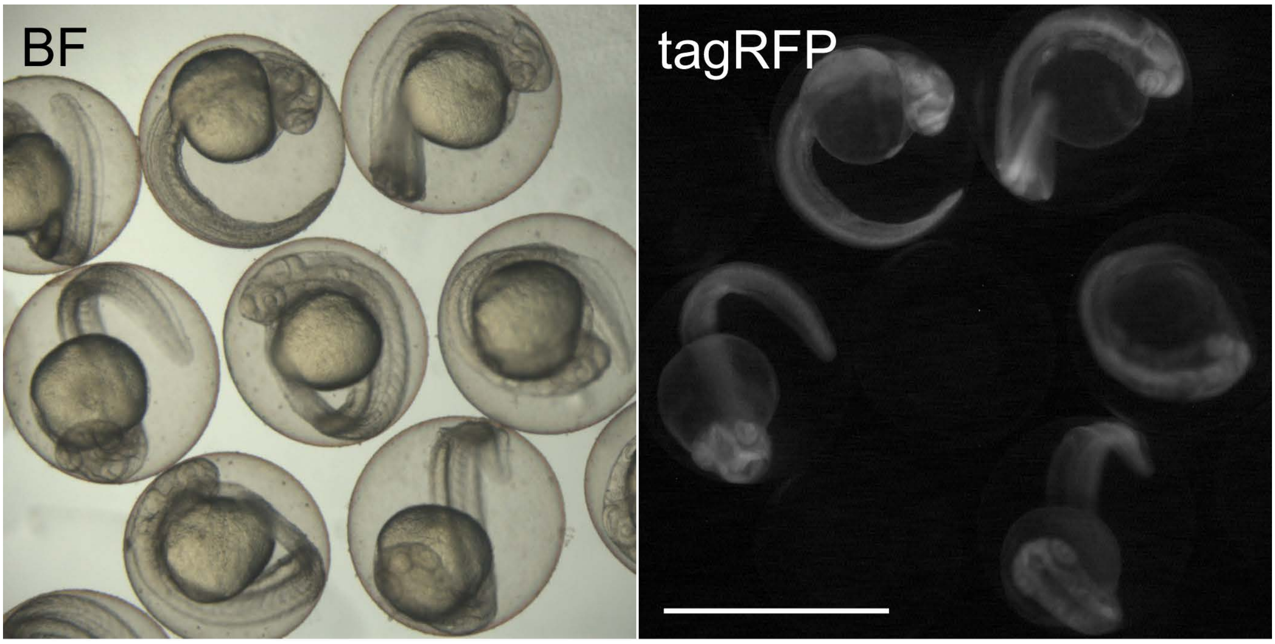

Supplementary Figure 1. Effect of chromobody induction by heat-shock in 24 hpf *hsp70l:PCNA-CB* transgenic embryos.
30 hpf embryos heat-shocked at 24 hpf. Ubiquitous fluorescence can be detected after induction. Transgenic embryos appear morphologically similar to non-transgenic siblings, indicating that chromobody expression is well tolerated *in vivo*. BF: brightfield. Scale bar: 1 mm.

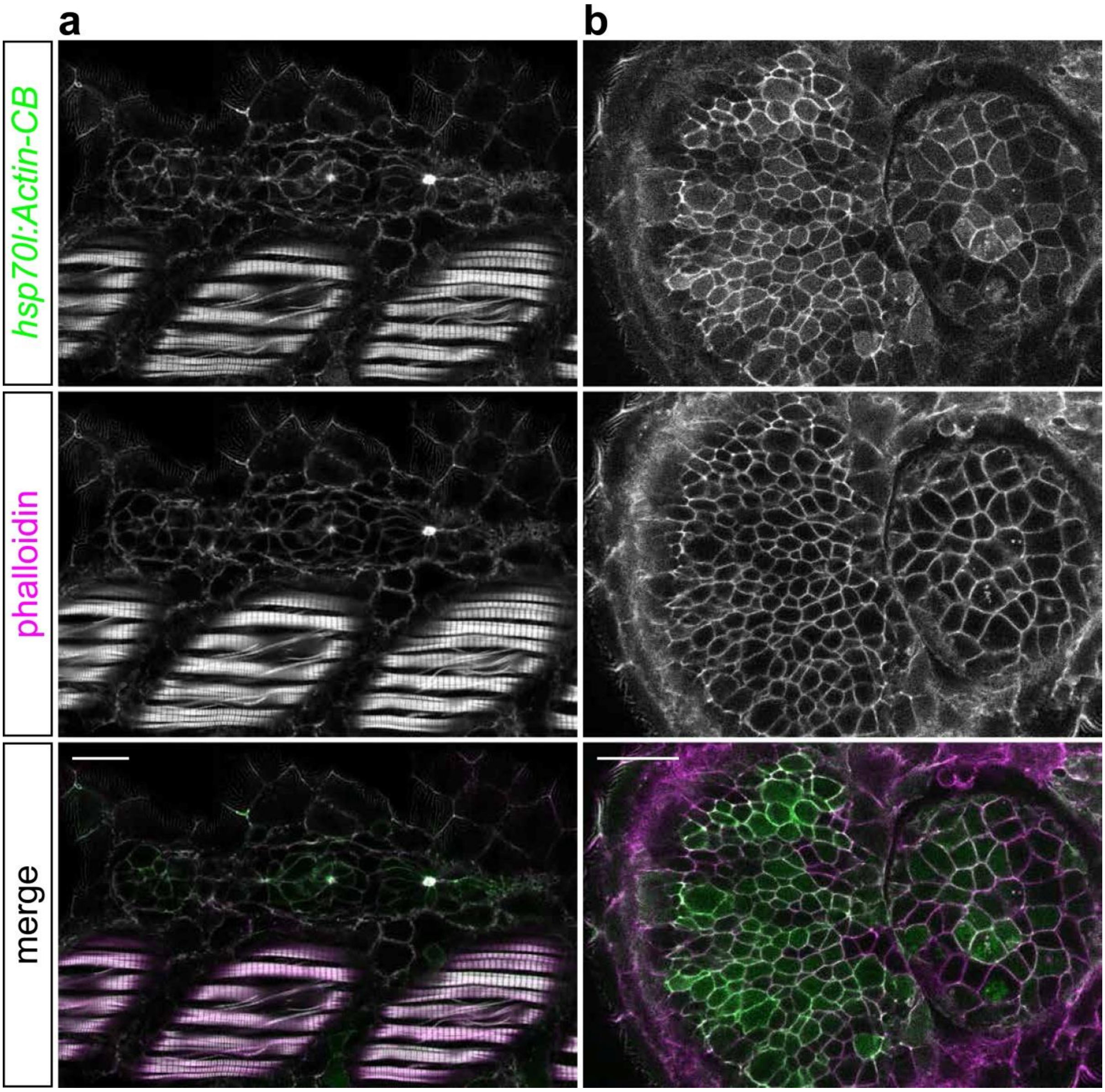

Supplementary Figure 2. Actin-CB colocalizes with zebrafish F-actin *in vivo*.
36 hpf *hsp70l:Actin-CB* embryos heat-shocked at 24 hpf and counterstained with rhodamine phalloidin. **(a)** Actin-CB fluorescence overlaps F-actin staining in muscle fibres, epidermal cells and in the apically-constricted centres of pLLP rosettes (similar to what has been described in Xu et al., 2014). **(b)** Actin-CB recognized cortical F-actin in cells of the retina and lens. Scale bars: 20 µm.

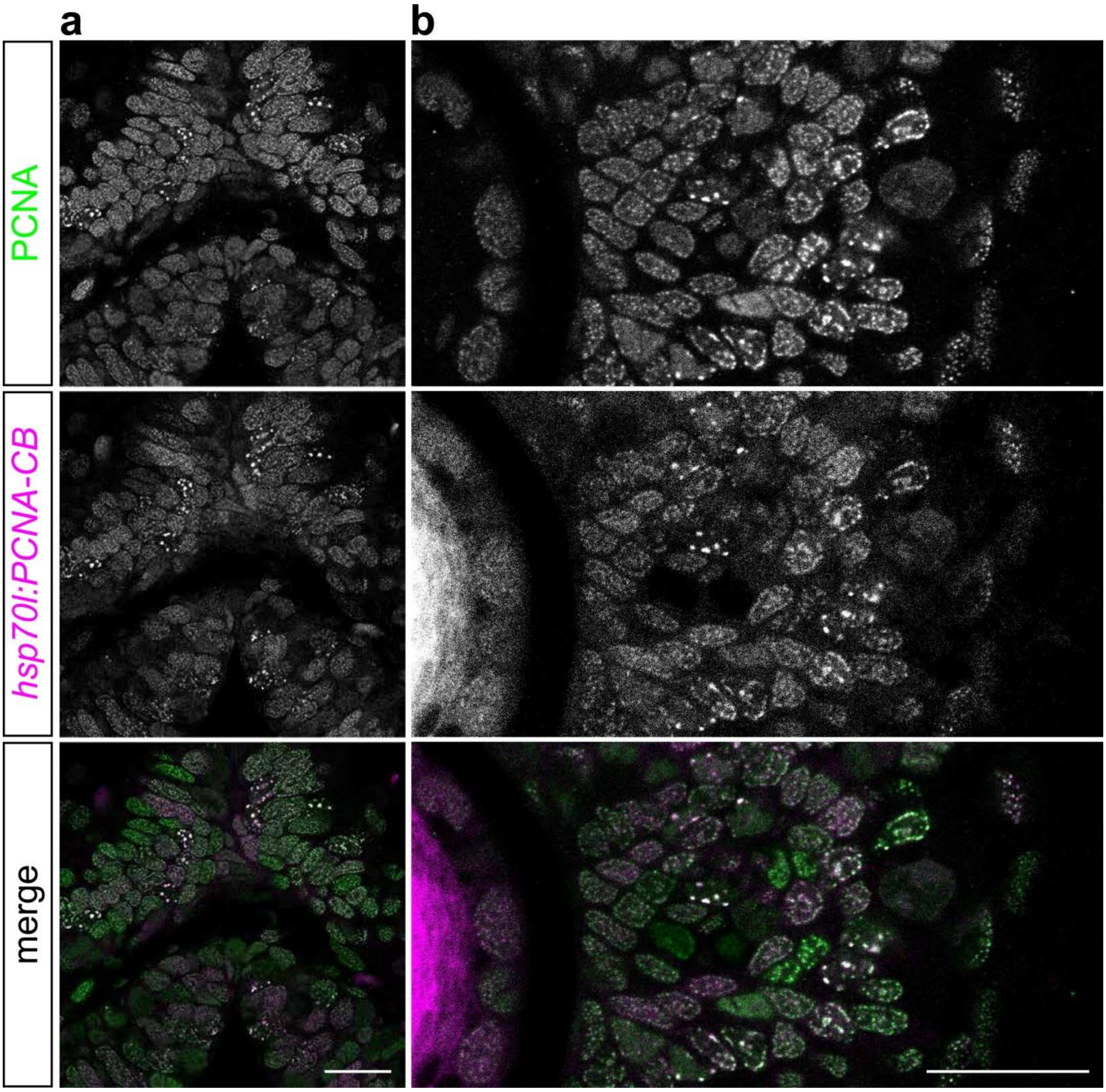

Supplementary Figure 3. PCNA-CB colocalizes with endogenous zebrafish PCNA *in vivo*.

36 hpf *hsp70l:PCNA-CB* embryos heat-shocked at 24 hpf and counterstained using an anti-PCNA antibody. PCNA-CB localizes to nuclear structures that contain endogenous PCNA protein in the dorsal midbrain **(a)** and retina **(b)**. Scale bars: 20 µm.

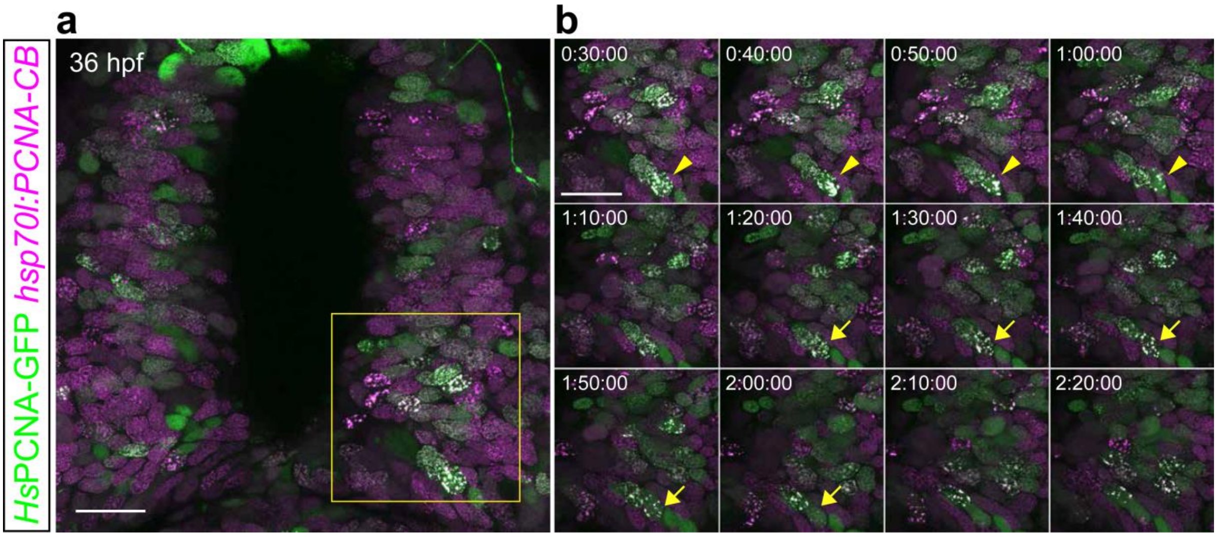

Supplementary Figure 4. PCNA-CB and human PCNA-GFP dynamically colocalize when expressed in live zebrafish.

36 hpf *hsp70l:PCNA-CB* embryos transiently expressing human PCNA-GFP from an injected DNA construct. **(a)** Overview of the scanned area. **(b)** Detailed time lapse analysis. Live imaging of animals heat-shocked at 24 hpf shows that PCNA-CB and human PCNA-GFP mark the same nuclear structures and their pattern is characteristic of S phase. Additionally, both fluorescent fusions can trace these structures' dynamics (**arrowheads** and **arrows** indicate two different cells over time). Scale bars: 20 µm. Timestamps: h:min:s.

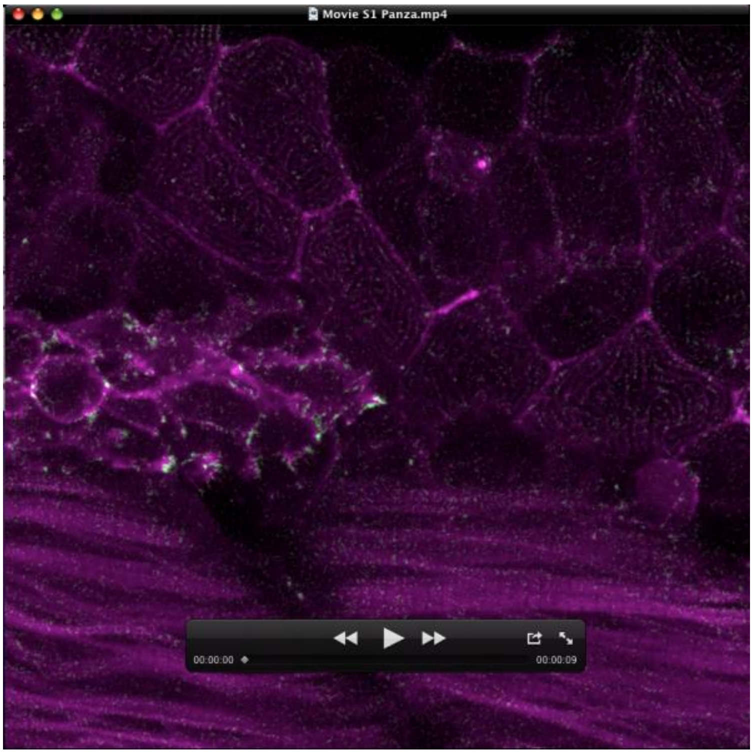

Supplementary Movie 1. Actin dynamics at the leading edge of posterior lateral line primordium (pLLP) cells during migration.

Time-lapse video of the migrating lateral line primordium in a 36 hpf *hsp70l:Actin-CB* embryo. Fast actin reorganization can be traced in cells at the posterior and lateral margin of the primordium. Magenta: signal from time frame *t*+1. Green: signal obtained from the subtraction of frame *t* from *t*+1. Green signal highlights the appearance of actin-CB signal from frame to frame.

Total duration of imaging: 22 min 30 s. Interval between consecutive frames: 7 s.

Objective: Zeiss LD C-Apochromat 40x/1.1 W Korr M27. Zoom: 2x. Pixel size: 0.107 µm.

Dimensions: 996x996 pixels.

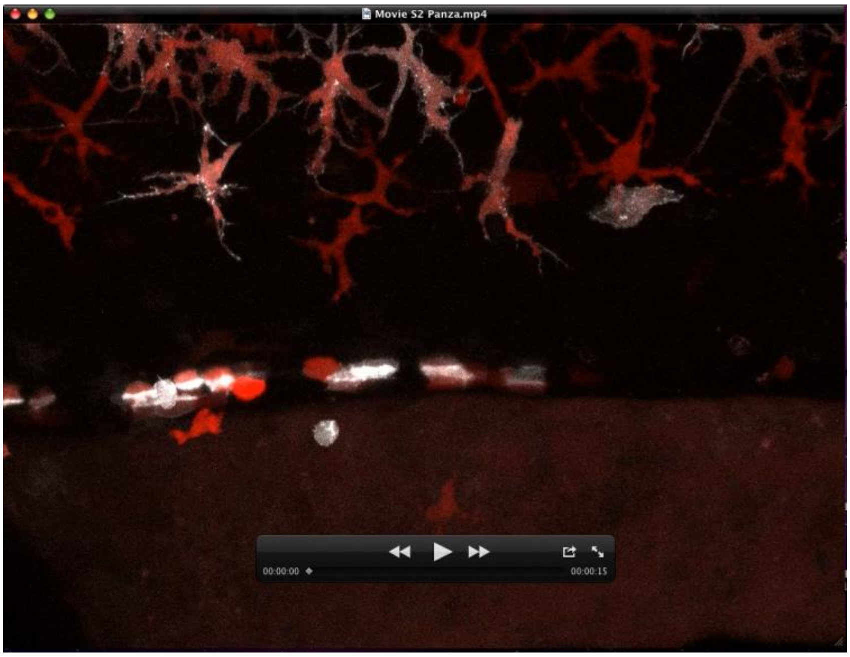

Supplementary Movie 2. Remodelling of actin-based cytoskeletal elements in embryonic xanthophores.

Extension and retraction of actin-rich filopodial projections in xanthophores of a *csf1ra:gal4*;*UAS:NTR-mcherry* (red); *UAS:Actin-CB* (white) embryo. Imaging was conducted from 30 hpf to 40.5 hpf with 8 min intervals.

Objective: Zeiss LD C-Apochromat 40x/1.1 W Korr M27. Zoom: 0.6x. Pixel size: 0.346 µm. Dimensions before editing: 1024x1024 pixels.

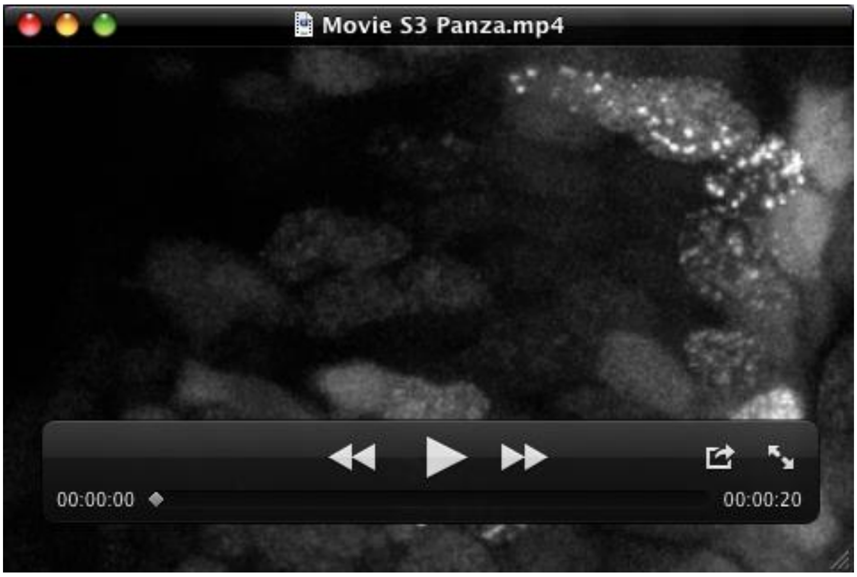

Supplementary Movie 3. Following endogenous PCNA localization shifts during cell cycle progression using chromobodies.

Detail from the dorsal midbrain of a *wnt1:gal4*;*UAS:PCNA-CB* (white) embryo. The localization dynamics of endogenous PCNA is traced by fluorescent chromobodies and mirrors the reported patterns described in mammalian cells (Leonhardt et al., 2000; Essers et al., 2005).

Imaging was carried out from 30 hpf to 43.3 hpf with 10 min intervals.

Objective: Zeiss LD C-Apochromat 40x/1.1 W Korr M27. Zoom: 0.8x. Pixel size: 0.259 µm. Dimensions before editing: 1024x1024 pixels.

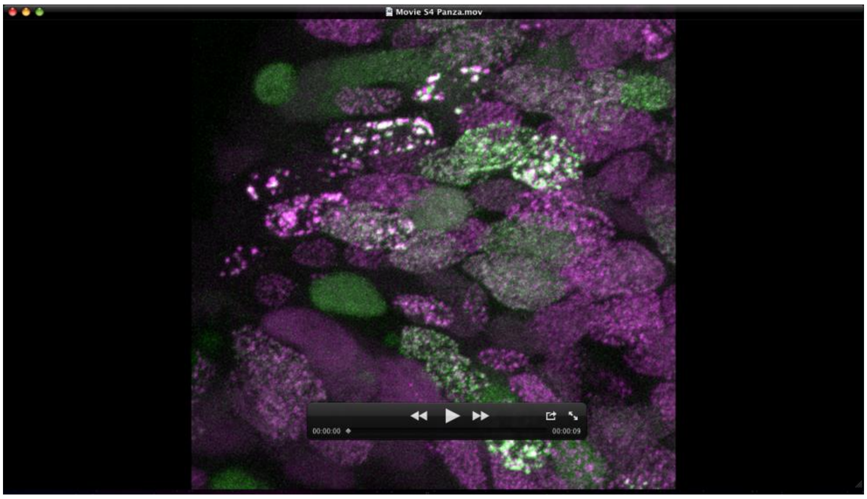

Supplementary Movie 4. Comparison between PCNA-CB and human PCNA-GFP during S phase progression.

Detail from the dorsal midbrain of a *hsp70l:PCNA-CB* embryo after transgenic expression (magenta) was induced at 24 hpf. This animal additionally shows expression of exogenous human PCNA-GFP (green) from an injected plasmid. The two fusion proteins dynamically colocalize in nuclear foci during S phase.

Imaging was carried out from 36 hpf to approximately 39 hpf with 10 min intervals.

Objective: Zeiss LD C-Apochromat 40x/1.1 W Korr M27. Zoom: 1.5x. Pixel size: 0.105 µm.

Dimensions before editing: 1348x1348 pixels.

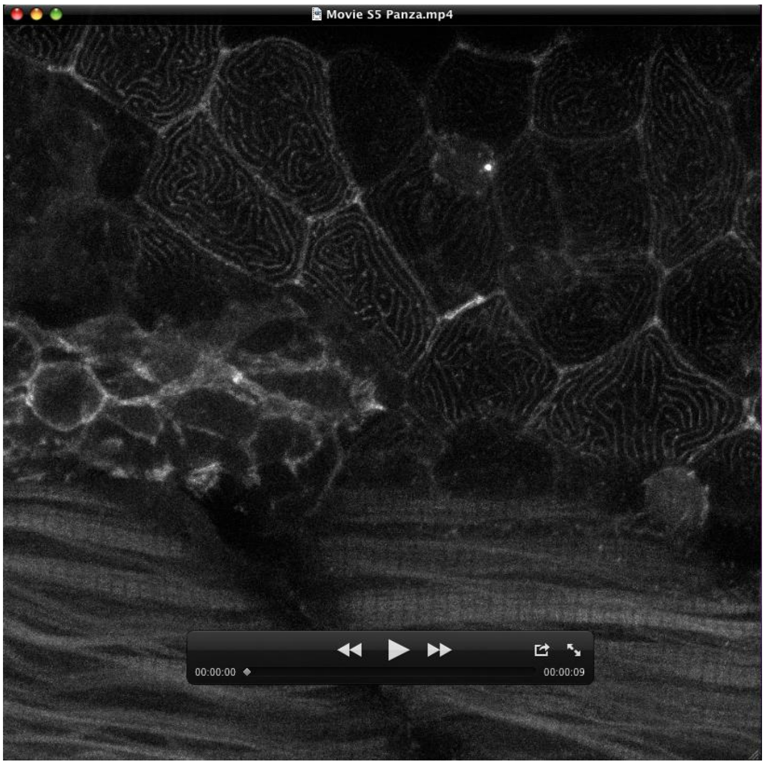

Supplementary Movie 5. As in Supplementary Movie 1, unprocessed.

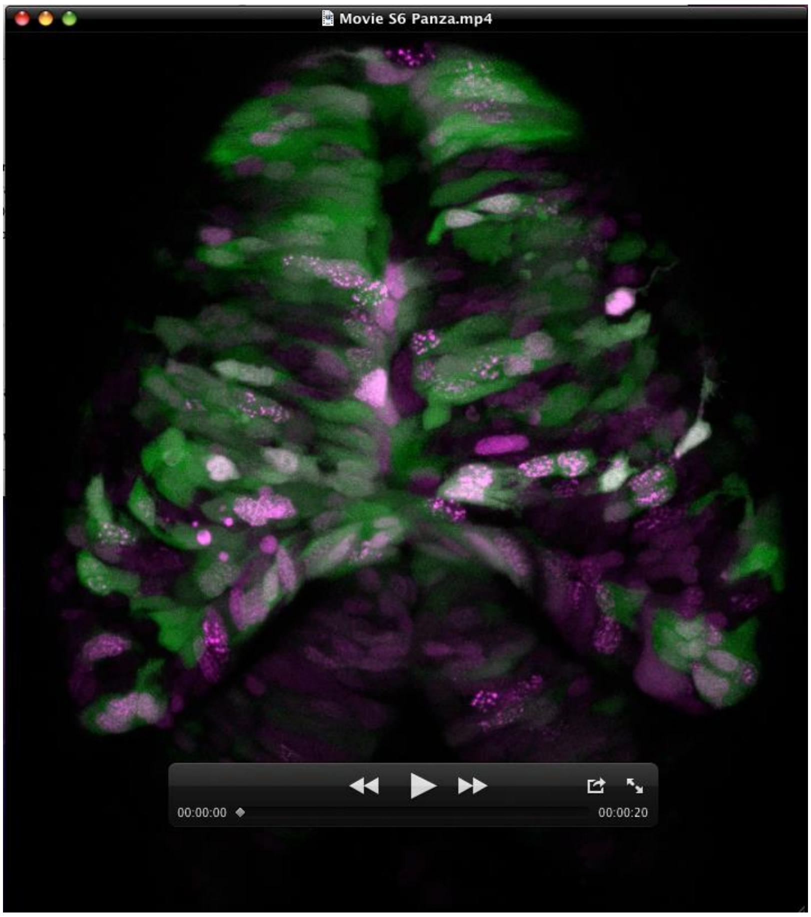

Supplementary Movie 6. As in Supplementary Movie 3, unprocessed.
